# Fluoxetine and Ketamine Reverse the Depressive but Not Anxiety Behavior Induced by Lesion of Cholinergic Neurons in the Horizontal Limb of the Diagonal Band of Broca in Male Rat

**DOI:** 10.3389/fnbeh.2021.602708

**Published:** 2021-02-18

**Authors:** Linghong Chen, Yuting Ke, Hong Ma, Lei Gao, Yiying Zhou, Huaqiang Zhu, Huifen Liu, Fuqiang Zhang, Wenhua Zhou

**Affiliations:** ^1^Zhejiang Provincial Key Laboratory of Addiction, Ningbo Kangning Hospital, School of Medicine, Ningbo University, Ningbo, China; ^2^Laboratory of Behavioral Neuroscience, Ningbo Kangning Hospital, Ningbo, China; ^3^School of Pharmacy, Shanghai Jiao Tong University, Shanghai, China; ^4^Department of Neurology, Massachusetts General Hospital and Harvard Medical School, Charlestown, MA, United States

**Keywords:** antidepressants, SSRI, choline acetyltransferase, rodent, ketamine, cholinergic neuron, basal forebrain

## Abstract

The basal forebrain cholinergic system is involved in cognitive processes, but the role of the basal forebrain cholinergic system in depression is unknown. We investigated whether a lesion of cholinergic neurons in the horizontal limb of the diagonal band of Broca (HDB) produces depressive-like behavior and whether fluoxetine or ketamine inhibits such depressive-like behaviors. Here, in rats, we used 192 IgG-saporin to eliminate the cholinergic neurons of the HDB and evaluated depressive-like behaviors using a preference test for sucrose solution and the forced swimming test. Fourteen days after the injection of 192 IgG-saporin into the HDB, the rats exhibited a significantly fewer number of choline acetyltransferase positive cell density in HDB, accompanied with neuronal loss in the entire hippocampus. Meanwhile, these rats significantly reduced preference for sucrose solution, increased immobility time in the forced swimming test, reduced locomotor activity, decreased context dependent memory in fear conditioning and the time spent in the open arms of the plus-maze. A single dose of ketamine (10 mg/kg) increased the sucrose solution consumption, reduced the immobility time in the forced swim test (FST), and increased locomotor activity compared to vehicle-treated rats. Moreover, in rats that were continuously treated with fluoxetine (10 mg/kg/day for 11 days), the sucrose solution consumption increased, the immobility time in the FST decreased, and locomotor activity increased compared to vehicle-treated rats. The present results demonstrate that a lesion of HDB cholinergic neurons results in depressive-like and anxiety-like behaviors and that antidepressants such as fluoxetine or ketamine, can reverse these depressive-like behaviors but not anxiety-like behaviors, and suggest that a lesion of HDB cholinergic neurons and followed hippocampus damage may be involved in the pathogenesis of depression.

## Introduction

The basal forebrain cholinergic complex, comprising the medial septum, the horizontal and vertical diagonal band of Broca (DB), and the nucleus basalis of Meynert, provides the major sources of cholinergic projection neurons to neocortex, hippocampus, and amygdala (Schliebs and Arendt, 2011; Ballinger et al., 2016). Cholinergic neurons in the basal forebrain regulate attention, learning, and memory by releasing acetylcholine to modulate neuronal processing in a wide range of forebrain areas (Wenk et al., 1997; Hasselmo, 1999; Yu and Dayan, 2005) and have been assumed to undergo moderate degenerative changes during aging, which has been related to the progression of memory deficits with aging (Teipel et al., 2005; Grothe et al., 2013). The horizontal diagonal band of Broca (HDB), densely innervates the main olfactory bulb (MOB), the first processing station within the main olfactory system (Case et al., 2017). Numerous studies suggest that removal of the olfactory bulbs results in numerous alterations in behavioral changes such as depressive behaviors in rodents (Morales-Medina et al., 2017). However, the role of HDB in depression still is unknown.

Brain imaging studies have revealed a reduction in the volume of limbic brain regions and other regions implicated in depression, such as the hippocampus and prefrontal cortex (Duman and Li, 2012). In addition, chronic stress exposure also causes neuron atrophy in the rodent hippocampus and prefrontal cortex (McEwen, 1999; Vyas et al., 2002; Banasr et al., 2007). The hippocampus in mammals receives massive input from the cholinergic neurons in the horizontal and vertical diagonal band of Broca. Given the strong connections between the medial septum-diagonal band of Broca (MSDB) and the hippocampus, damage to the hippocampus generally produces similar effects as MSDB dysfunction (Gray and McNaughton, 1983). Thus, we hypothesized that a lesion of the HDB may produce depressive-like behaviors in rat.

The aim of the present study was to investigate whether a lesion of HDB cholinergic neurons will produce depressive-like symptoms in rodent behavioral despair tests, including the forced swim test (FST), and the anhedonia, a core symptom of depression. On the other hand, we also evaluated the anxiety-related behavior and Pavlovian learning assessed by the elevated plus maze (EPM) and the fear conditioning test (FCT) paradigm. Here, we used 192 IgG-saporin, which is a conjugate of an antibody against the nerve growth factor receptor and the ribosomal toxin saporin, which after penetration into the cell inactivates ribosomes and arrests protein synthesis, resulting in loss of cholinergic neurons (Walsh et al., 1995; Wrenn and Wiley, 1998; Cooper-Kuhn et al., 2004). Fluoxetine, a selective serotonin reuptake inhibitor (SSRI), is the commonly prescribed treatment for depression. Chronic treatment with fluoxetine was shown to produce an antidepressant-like effect in the FST with mice (Holick et al., 2008), prevent or reverse effectively stress-induced changes, and promote several forms of neuronal plasticity (Malberg and Duman, 2003; Siopi et al., 2016). Moreover, ketamine produces rapid antidepressant responses (within hours) in treatment resistant major depressive disorder (MDD) patients (Berman et al., 2000; Autry et al., 2011). Preclinical studies have demonstrated the antidepressant actions of ketamine in rodent behavioral despair tests, including the FST and the learned helplessness paradigm (Li et al., 2011; Duman and Li, 2012), which is involved in increasing α-amino-3-hydroxy-5-méthylisoazol-4-propionate receptor (AMPAR) activity, levels of phosphorylated mechanistic target of rapamycin (mTOR), and expression of brain-derived neurotrophic factor (BDNF) (Ignacio et al., 2016). Here, we observe whether selective lesion of HDB would produce depressive-like behaviors in rats and test whether the depressive-like behaviors induced by an HDB lesion will be reversed by chronic treatment with fluoxetine or acute treatment with ketamine.

## Materials and Methods

### Animals

Adult male Sprague-Dawley rats (8 weeks old, *n* = 78) with body weight of 250–300 g purchased from the Experimental Animal Center of Zhejiang Province (Hangzhou, China). Animals were acclimated to the facility for 1 week prior to beginning the experiments. Rats were housed in groups of 3–5 per standard rat cage, at least 2 weeks before the experiments. The animals were maintained in a temperature- and humidity-controlled room with a reversed 12-h light/dark cycle (light onset at 19:00 h, offset at 07:00 h), with food and water available *ad libitum*. The rats were singly housed to avoid the aggressive fighting after surgery. All experimental procedures in this study were carried out in accordance with the National Institutes of Health Guide for the Care and Use of Laboratory Animals (NIH publications No. 80-23, revised 1996). The study was also approved by the Center’s ethics committee [approval no. SYXK (Zhe) 2013-0192].

### Stereotaxic Surgery and Infusions

Rats were anesthetized with sodium pentobarbital (50 mg/kg, i.p.) and placed in a stereotaxic apparatus with bregma and lambda in a horizontal plane. A stainless-steel cannula (5-μL Hamilton syringe) was inserted bilaterally into the HDB using an AP (from bregma) of +0.48 mm, a ML (from the midline) of ±0.8 mm and a DV (from the brain surface) of -8.0 mm (Paxinos and Watson, 1998). Injections of 192 IgG-saporin (Millipore, Billerica, MA, United States) were performed in a volume of 0.5 μL per side with a concentration of 0.5 μg/μL according to previous reports (Cutuli et al., 2013; Kalinchuk et al., 2015). After each injection, the needle was left in place for 5 min before being slowly retracted. In all sham-operated rats, a volume of 0.5 μL of PBS was injected into each side. In all cases, 192 IgG-saporin or PBS were injected using a microdialysis pump at a flow rate of 0.1μL/min. At the end of injections, the probe was left in place for additional 5 min. The incision was sutured, and animals were placed onto a warm heating pad during recovery. Following surgery, the rats received 0.4 mL saline containing a 50 IU penicillin B (i.m.) each day for 3 days to prevent potential bacterial infections.

### Drugs

Fluoxetine hydrochloride (FLX) was purchased from Sigma-Aldrich (St Louis, MO) and dissolved in DMSO and diluted in 0.9% sterile saline (NaCl) with a final concentration of 1% DMSO. Ketamine hydrochloride was purchased from the Hengrui Pharmaceutical Co., Ltd. (Jiangsu, China) and dissolved in 0.9% sterile saline (NaCl). The dose of ketamine or FLX at 10 mg/kg was chose as described before and was administered intraperitoneally (i.p.) (Garcia et al., 2008; Fernandez Macedo et al., 2013). Control animals received same amount of 0.9% sterile saline i.p. or vehicle i.p. (DMSO 1%).

### Experimental Design

In order to assess whether a lesion of the cholinergic neurons in the HDB produces depressive-like behavior and FLX or ketamine inhibits such depressive-like behavior, three sets of experiments were conducted according to the following experimental design. The sequence of the behavioral tests was described in previous studies with minor modifications (Cutuli et al., 2013; Ke et al., 2019; Dao et al., 2020). *Experiment 1* (Figure 3A): Rats were randomly divided into three groups (*n* = 10 rats per group): non-operated (Control), sham operated (Sham) and 192 IgG-saporin injection (Ig-saporin). Two weeks after the surgery, rats were introduced to five tests in the sucrose preference test (SPT) on day 14, locomotor activity test (LAT) on day 19, the elevated plus maze (EPM) test on day 21, the forced swim test (FST) on day 23 and the fear conditioning test (FCT) on day 27. *Experiment 2* (Figure 4A): Rats were randomly divided into three groups (*n* = 6 rats per group): Sham + saline (Sham + sal), Ig-saporin + saline (Ig-saporin + sal), and Ig-saporin + ketamine (Ig-saporin + ket). Two weeks after the surgery, the ketamine treated rats were administered with ketamine (10 mg/kg, i.p.) once at the beginning of the test on day 14. The two other groups received the same amount of saline (i.p.). To assess the antidepressant effect of ketamine, rats were tested SPT on day 15, 17, and 25, LAT on day 19, EPM on day 21, and FST on day 23. *Experiment 3* (Figure 5A): rats were divided into three groups (*n* = 6 rats per group): sham + vehicle (Sham + veh), Ig-saporin + vehicle (Ig-saporin + veh), and Ig-saporin + FLX. Two weeks after the surgery, the FLX treated rats were administered with FLX (10 mg/kg, i.p.) daily injections at the beginning of the test starting on day15 and continuously received the daily treatment of FLX throughout till the end of the last behavioral test. Two other groups received the same amount of vehicle (1% DMSO diluted in saline, i.p.). To assess the antidepressant effect of FLX, rats were tested SPT on day 15, 17, and 25, LAT on day 19, EPM on day 21 and FST on day 23. Behavioral tests were performed between 1 P.M. and 7 P.M. Rats were moved to the testing room to acclimatize 3 h before the test. Each test was separated by at least 48 h to prevent interference with the others. In all behavioral tests, experimenters were blinded to animal groups during the tests and data analysis.

### Sucrose Preference Test

Sucrose preference test (SPT) was used to evaluate depressive-like behavior (Willner et al., 1987), rats were trained to adapt to 1% sucrose solution (w/v) for 2 days, they were given one bottle of 1% sucrose solution and one bottle of tap water, with the position of the two bottle exchanged every 12 h. After adaptation, the rats were deprived of water and food for 24 h, followed by SPT, in which rats were housed in individual cages, performed between 1:00 P.M. and 2:00 P.M and had free access to two bottles, one containing 1% sucrose and the other containing tap water. After 1 h, sucrose and water consumption (mL) were measured, and sucrose preference was calculated as sucrose preference (%) = sucrose consumption/(sucrose consumption + water consumption).

### Locomotor Activity Test

Locomotor activity test (LAT) was based on a previous study (Kelly et al., 1998). Each rat was put into a 100 × 100 × 40 cm open-field chamber that was enclosed in a soundproof box and was housed in a quiet room. A video camera which was connected to a computer was located over the box. Each rat was placed gently in this novel and empty arena, allowed to explore freely for 1 h. Horizontal distance was measured by infrared photobeam sensors and analyzed by t SMART video tracking system(MobleDatum, China). Movement time was recorded when a rat was active for >1 s. All of tests were performed between 1:00 P.M. and 6:00 P.M. After each test, the apparatus was cleaned with a 70% ethanol solution.

### Elevated Plus Maze

The elevated plus maze (EPM) test was widely used to assess the anxiety-like behavior (Iniguez et al., 2010). The EPM was made of two opposing open arms [50 (L) × 10 (W) cm] and two opposing closed arms [50 (L) × 10 (W) × 40 (H) cm] connected by a central platform [10 (L) × 10 (W) cm]. The maze was elevated to a height of 70 cm with a video camera placed on top of it. Each rat was placed gently into the center square facing one of the open arms, and allowed to explore the maze for 5 min. The time spent in the open arms and numbers of entries into the open and closed arms was recorded by infrared photobeam sensors and analyzed by SMART video tracking system (MobileDatum, China). A ratio of time spent in open arms divided to time in open + closed arms, and entries to open arms divided to entries into open + closed arms were used as anxiety measures. After each test, the maze was cleaned with a 70% ethanol solution. All tests were performed between 1:00 P.M. and 5:00 P.M. and carried out in a noise-, temperature-, and light-controlled room.

### Fear Conditioning Test

Fear conditioning test (FCT) was used for evaluation of fear memory and learning performance as previously study described (Zhang et al., 2016). The apparatus for FCT was consisting of a white Plexiglas chamber [40 (L) × 40 (W) × 50 (H) cm] with grid floor (stainless steel rods); each rod was 5 mm diameter and spaced 1 cm apart. The chamber placed inside a sound attenuation box with an illuminated white light during the test and 65 dB background noise generated by the fan inside. The day before the testing day, each rat was placed into the chamber and allowed to explore freely for 2 min. A 2 s electric foot-shock with 1 mA intensity was then delivered at the end of the tone, as the unconditioned stimulus (US). The presentation of US repeats three times at 60 s interval. After 30 s of the last shock, rats were immediately returned to their home cage and chambers were wiped with 70% ethanol between each test. After 24 h, rats were returned to their previous chambers with the same context for 5 min without any tone or foot shock. The context-dependent freezing time in the rats was recorded by a video on the top of the chamber and the data was analyzed by video freeze software (Med Associates). After each test, the chamber was cleaned with a 70% ethanol solution.

### Forced Swim Test

The forced swim test (FST) consisted of 2 days with sessions lasting 15 and 5 min, respectively (Brenes et al., 2008; Ke et al., 2019). Briefly, rats were individually placed in a clear Plexiglas tank (height: 40 cm; diameter: 30 cm) filled to 21.5 ± 1.5 cm with fresh water at 24 ± 0.5°C for a 15 min pre-exposure on the training day. On the testing day, rats were placed in the same tank and videotaped for 5 min. The immobility time during the total 5 min test, which is defined as a lack of motion of the whole body except for small movements that are necessary to keep the head above water, was scored by observers that were blind to the groups. Immobility time was defined when rats remained floating or motionless with only little movements which were necessary for keeping balance in the water. After each test, rats were removed from the water, dried with paper towels and placed in a dry cage warmed with a 300 W lamp on bottom of cage until the animal was totally dry, and then returned to their home cage. The water in the tank was renewed between each animal. All tests were performed between 1:00 P.M. and 5:00 P.M.

### Brain Tissue Fixation

After all of the behavioral tests were completed, rats were deeply anesthetized with sodium pentobarbital (50 mg/kg, i.p.) and were perfused through the heart with 250 mL of 0.9% sterile saline followed by 200 mL of a 4% paraformaldehyde solution in 0.1 M phosphate buffer (PB), pH 7.4. The rat brain was removed and post-fixed with a 4% paraformaldehyde solution in 0.1 M PB, pH 7.4 at 4°C overnight. These brains were then transferred to 30% sucrose in 0.1 M PB, pH 7.4, at 4°C for further use.

### Hematoxylin and Eosin Staining

Hematoxylin and Eosin (H&E) staining was carried out to examine cellular morphology by observing the number and shape of the neurons. Using a cryostat (Leica CM1950), the brains were cut into 6–8 μm thick coronal sections that were collected on gelatin-coated slides. The sections were dried at room temperature for at least 1 h and stained using H&E according to the protocol. Dehydrate the slides in ascending alcohol solutions and cleared with xylene twice in staining dishes. Mount coverslip onto the section on glass slide with Permount.

### Immunohistochemistry

The degree of destruction of cholinergic fibers in the HDB were assessed by choline acetyltransferase (ChAT) immunocytochemistry. The procedures were performed as previously described. Briefly, 30 μm thick coronal brain sections were cut by a cryostat (Leica CM1950). The floating sections were rinsed in PBS, blocked in PBS containing 5% normal goat serum, 1% BSA, 0.03% H_2_O_2_, 0.2% triton X-100 at room temperature for 1 h, and then incubated in a primary antibody solution containing rabbit polyclonal ChAT (1:400; Abcam, United Kingdom) in 5% normal goat serum, 1% BSA, 0.2% triton X-100 at 4°C for 48 h. After washing three times with PBS, the sections were incubated at 37°C with peroxidase-conjugated goat anti-rabbit IgG (1:200; PK-4001; Vector Laboratories) for 2 h. After rinsing four times in PBS, the sections were incubated in an ABC complex solution (Vector Laboratories) for 2 hat 37°C and enhanced in a solution containing 50% diaminobenzidine tetrahydrochloride (DAB, Sigma) and 0.1% hydrogen peroxide in PBS at room temperature. Finally, all sections were dehydrated, cleared and mounted with Permount, and photographed using a BX41 microscope (Olympus, Shinjuku-ku, Tokyo, Japan). Immunostaining was quantified by digital image analysis with Image-Pro Plus 6.0 software. ChAT immune-positive (cholinergic) neuronal cell bodies in the horizontal and vertical diagonal band of Broca area were counted on the pictures. Individuals who were blind to the treatment of the rats performed the cell counts.

### Statistics

Statistical analysis was performed using GraphPad Prism^§^ v8.0 (GraphPad Software, La Jolla, CA, United States), and the accepted significance level for all tests was *P* < 0.05. All data were expressed as the mean ± S.E.M. Two-tailed unpaired Student *t*-test was used to compare the differences of ChAT neurons and hippocampus cells between two groups, and one-way ANOVA followed by Tukey *post-hoc* test was used for multiple comparisons in the HDB-cholinergic lesion rats. Non-parametric inferential tests were used for behavioral tests in ketamine and FLX treated rats, the Kruskal-Wallis one-way analysis of variance was used to compare three groups followed by Dunn’s *post-hoc* test for multiple comparisons.

## Results

### Selective Elimination of Cholinergic Neurons in the HDB by 192 IgG-Saporin

**FIGURE 1:**
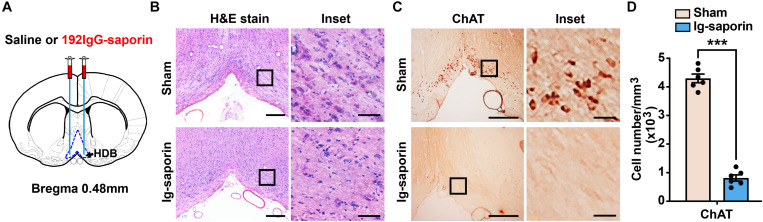
Infusion of 192 IgG-saporin into the HDB substantially reduced the number of cholinergic neurons. **(A)** Location of cannula placements in the HDB. **(B)** H&E stain showed the cell loss in the HDB. **(C)** ChAT immunostainings in the HDB showed a near complete loss of cholinergic neurons. **(D)** Quantitative analysis revealed significant differences in the number of ChAT-positive cell density between the two groups (*n* = 6 per group). Data are represented as mean ± SEM; ***p < 0.001, compared to the sham group; two-tailed *t*-test. Scale bars: 300 μm; 50 μm (inset).

One hundred ninety-two IgG-saporin is an immunotoxin that is directed against the rat p75 nerve growth factor receptor, a low-affinity neurotrophin receptor (Boskovic et al., 2019). The cholinergic neurons of the basal forebrain specifically express p75 nerve growth factor receptor, and injecting 192 IgG-saporin into the HDB results in cholinergic cell loss and a depletion of ChAT activity in the HDB (Figure 1A). After 2 weeks, to ensure that the cholinergic neuron lesions in this experiment were accurate and complete, we used H&E stain to show the cell loss in the HDB (Figure 1B) and analyzed the remaining cholinergic neurons of the HDB using ChAT immunoreactivity. The representative images are shown in Figure 1C, and near complete loss of the average number of ChAT-immunoreactive neurons in the HDB was observed in the group of rats lesioned with 192 IgG-saporin compared to the sham-lesioned rats that were infused with saline. Two-tailed unpaired Student *t*-test revealed significant differences in the number of ChAT-positive cell density between the two groups (Figure 1D). The 192 IgG-saporin treated group rats exhibited a significantly fewer number of ChAT-positive cell density compared to the sham group rats (*t* = 18.65, *P* < 0.001, *n* = 6 in each group).

### Hippocampus Cell Loss in the HDB-Cholinergic Lesions Rats

**FIGURE 2:**
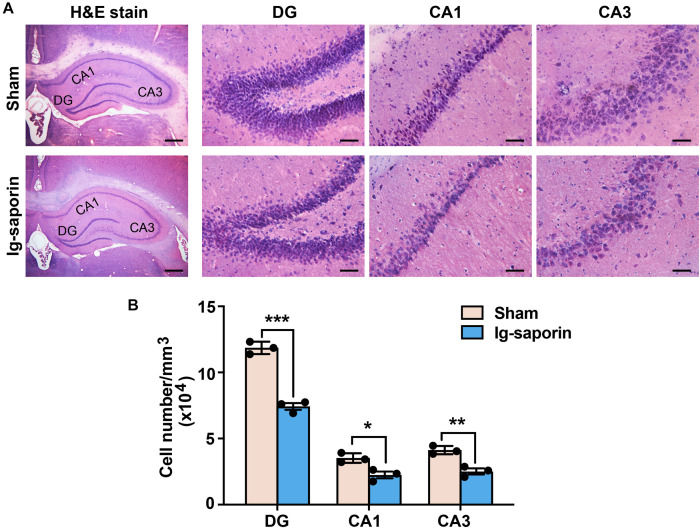
Effects of 192 IgG-saporin on the morphological alterations and toxicity in the dentate gyrus (DG) and CA3 and CA1 regions of the hippocampus. **(A)** H&E staining showed a reduce of the number of cells in the hippocampus. **(B)** Quantitative analysis revealed significant differences in the total number of pyramidal neurons between the two groups (*n* = 3 per group). Data are represented as mean ± SEM; *p < 0.05, **p < 0.01 and ***p < 0.001. Scale bars: 500 μm; 50 μm (inset).

The H&E staining results revealed a reduction of the local cell layers in the hippocampal CA1, CA3, and dentate gyrus (DG) regions (shown in Figure 2A). The hippocampal neurons in the sham group were compact compared to those in the 192 IgG-saporin treated group. As shown in Figure 2B, multiple *t*-test revealed significant differences between the two groups in the total number of pyramidal neurons in CA1 (*t* = 11.93, *P* < 0.001, *n* = 3 in each group), CA2 (*t* = 3.86, *P* < 0.05, *n* = 3 in each group) and CA3 (*t* = 5.54, *P* < 0.01, *n* = 3 in each group).

### Effects of HDB-Cholinergic Lesions on Depressive-Like Behaviors in Rats

**FIGURE 3:**
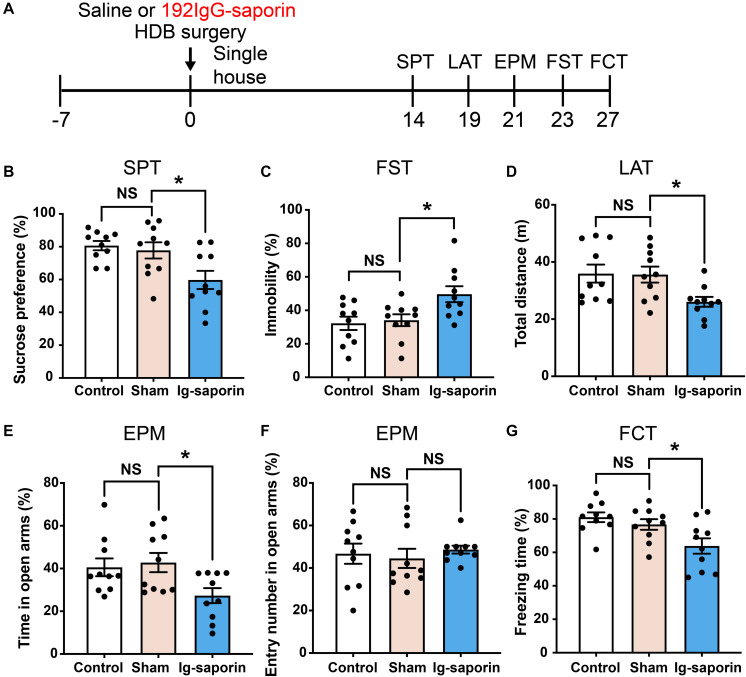
Effects of HDB-cholinergic lesions on depressive-like behaviors in rats. **(A)** Experimental schedules. The sucrose solution preference was significantly decreased **(B)**; immobility time in the FST significantly increased **(C)**; locomotor activity reduced **(D)**; in the EPM, the time spent in the open arms decreased **(E)**; no changes in the percentage of entries into the open arms **(F)**; and a short-term memory deficit in contextual fear conditioning **(G)** in the 192 IgG-saporin rats compared to the Sham group. The data are expressed as mean ± SEM (*n* = 10 per group). *p < 0.05, compared to Sham group. NS, not significant.

The influence of HDB-cholinergic lesions that were induced by using 192 IgG-saporin on depressive-like behavior in rats was shown in Figure 3. One-way ANOVA revealed significant differences among these groups in the SPT [*F*_(2_, _27)_ = 6.089, *P* = 0.007; Figure 3B]. *Post-hoc* test showed that the preference to sucrose solution was significantly decreased in the 192 IgG-saporin treated rats compared to the Control or Sham group (both *P* < 0.05). Similarly, there are significant differences of immobility time in the FST among the groups [*F*_(2_, _27)_ = 5.343, *P* = 0.011; Figure 3C], *post-hoc* test showed the increased immobility time in the 192 IgG-saporin treated group compared to the Control or Sham group (both *P* < 0.05). In the EPM, there are significant differences of the time spent in the open arms [*F*_(2_, _27)_ = 4.205, *P* = 0.026; Figure 3E], but no difference in the percentage of entries into the open arms among the groups [*F*_(2_, _27)_ = 0.276, *P* = 0.761; Figure 3F]. The time spent in the open arms decreased in the 192 IgG-saporin treated group compared to the Sham group (*P* < 0.05), but with no changes in the percentage of entries into the open arms compared to the Sham group (*P* > 0.05). In addition, significantly reduced locomotor activity is different significantly among the groups [*F*_(2_, _27)_ = 4.547, *P* = 0.019; Figure 3D], 192 IgG-saporin treatment significantly reduced locomotor activity compared to the Control or Sham group (both *P* < 0.05). Moreover, there are significant difference in the freezing time among the groups [*F*_(2_, _27)_ = 6.075, *P* = 0.007; Figure 3G], 192 IgG-saporin treated rats have a learning or short-term memory deficit in contextual fear conditioning. There was no significant difference between the control group and the Sham group in all behavioral tests.

### Ketamine Mitigates Depressive-Like Behaviors in HDB-Cholinergic Neuron Lesioned Rat

**FIGURE 4:**
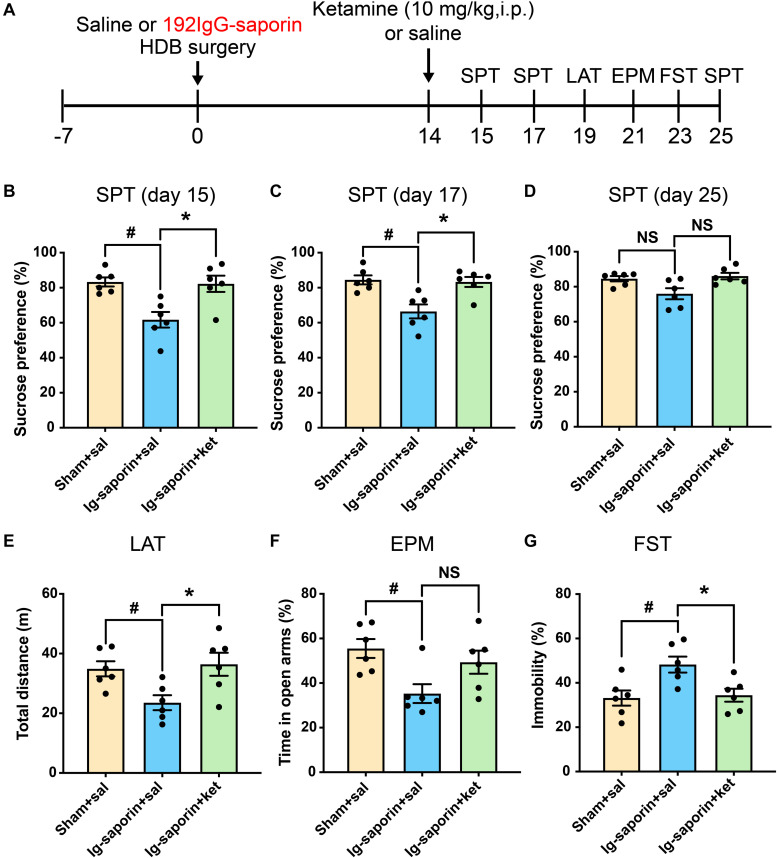
Effects of a single treatment of ketamine on depressive-like behaviors following HDB-cholinergic lesions in rats. **(A)** Schematic representation of the experimental design. After 2 weeks, acute ketamine treatment at a dose of 10 mg/kg significantly increased sucrose consumption in the SPT on day15 **(B)**, on day17 **(C)**, and on day 25 **(D)**. In addition, acute ketamine administration significantly increased locomotor activity **(E)** and decreased immobility time in the FST **(G)**. In contrast, no significant differences were found between ketamine treatment and the vehicle group in the EPM **(F)**. Each bar represents the mean ± SEM (*n* = 6 per group). *p < 0.05 and ^#^*p* < 0.05; NS, not significant.

After 2 weeks, the effect of ketamine on the depressive-like behaviors induced by HDB-cholinergic lesions in rats was examined at 24 h after one-time injection. As shown in Figure 4, the Kruskal-Wallis statistics revealed the significant differences of sucrose consumption in the SPT on day15 (*P* = 0.0034; Figure 4B), on day 17 (*P* = 0.0056; Figure 4C), and on day 25 (*P* = 0.044; Figure 4D). *Post-hoc* test showed that sucrose consumption increased significantly on 1, 3 days after single treatment of ketamine compared to their vehicle treated group (all *P* < 0.05), and increased to a certain extent on 11 days after single treatment of ketamine but not significant difference between two groups (*P* = 0.085). Meanwhile, there were no difference between the ketamine treated group and Sham group (all *P* > 0.05). In addition, there are significant differences of immobility time in the FST (*P* = 0.0084; Figure 4G) and locomotor activity (*P* = 0.013; Figure 4E) among the groups. Multiple comparison showed that acute ketamine treatment significantly decreased immobility time in the FST and increased locomotor activity compared to the vehicle group (both *P* < 0.05). In contrast, even there was significant difference among the groups in the EPM on day 21 (*P* = 0.035; Figure 4F), but *post-hoc* analysis showed that there were no significant differences of time spent in open arms were found between ketamine treatment group and the vehicle group (*P* > 0.05, *n* = 6 in each group). There was no significant difference between the ketamine treated group and the Sham group in all behavioral tests.

### Fluoxetine Mitigates Depressive-Like Behavior in HDB-Cholinergic Neuron Lesioned Rat

**FIGURE 5:**
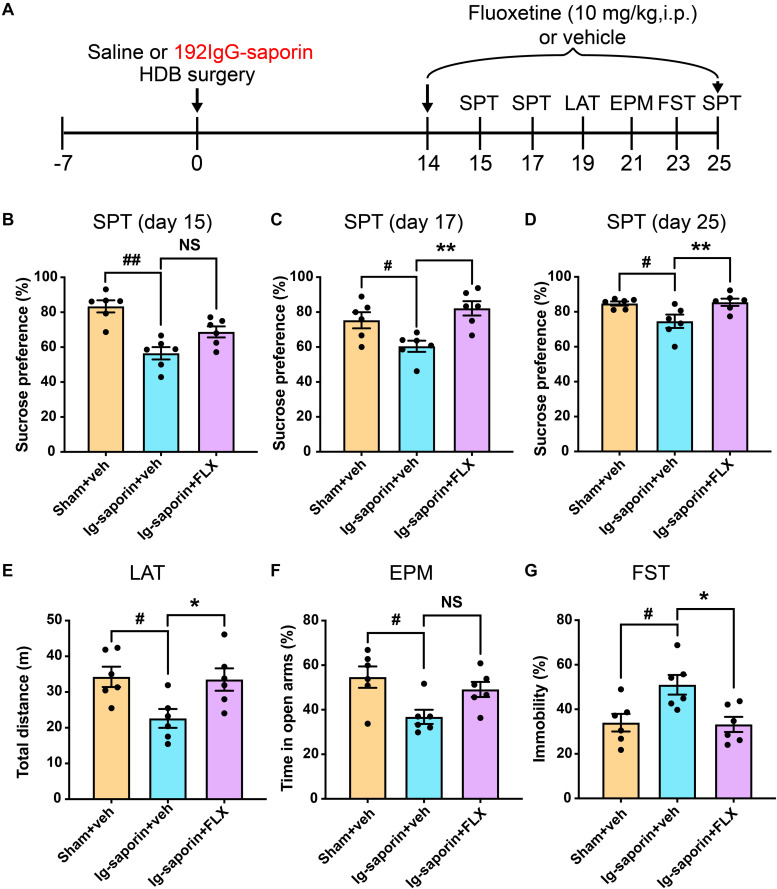
Effects of chronic treatment of FLX on depressive-like behaviors following HDB-cholinergic lesions in rats. **(A)** Schematic representation of the experimental design. The data revealed that first day treatment of FLX at the dose of 10 mg/kg had no effect on the sucrose consumption in the SPT on day 15 **(B)**, but continuous treatment with FLX increased significantly the sucrose consumption in the SPT on day 17 **(C)** and on day 25 **(D)** compared to the vehicle group. The chronic treatment with FLX increased locomotor activity **(E)** and decreased immobility time in the FST **(G)**. In contrast, no significant differences between FLX treatment and the vehicle group in the EPM **(F)**. Each bar represents the mean ± SEM (*n* = 6 per group). *p < 0.05 and **p < 0.01; ^#^*p* < 0.05 and ^##^*p* < 0.01; NS, not significant.

After 2 weeks, the effect of chronic treatment with FLX on the depressive-like behaviors induced by HDB-cholinergic lesions in rats was examined. As shown in Figure 5, the Kruskal-Wallis statistics showed that there was significant difference among these groups in the SPT day 15 (*P* = 0.0002; Figure 4B), but *post-hoc* analysis showed that acute treatment of FLX at the dose of 10 mg/kg has no effect on the sucrose consumption (*P* > 0.05), but after 3 days continuous administration of FLX (10 mg/kg, i.p.) significantly increased the sucrose consumption in rats on day 17 (*P* = 0.0012; Figure 5C) and chronic treatment with FLX for 11 days increased the sucrose consumption on day 25 (*P* = 0.001; Figure 5D). the Kruskal-Wallis statistics revealed the significant differences in the time of immobility in the FST (*P* = 0.013; Figure 5G) and locomotor activity (*P* = 0.0084; Figure 5E). The FLX chronic treated rats showed less immobility time in the FST and increased locomotor activity compared to the vehicle group (both *P* < 0.05). In contrast, even there was significant difference among three groups in the EPM day 21 (*P* = 0.031; Figure 5F), but *post-hoc* analysis showed that there were no statistical differences between FLX treatment group and the vehicle group (*P* > 0.05). There was no significant difference between the FLX treated group and the Sham group in all behavioral tests.

## Discussion

In the present study, the HDB cholinergic neurons were lesioned by using 192 IgG-saporin and its effect on depressive-like behaviors was investigated. The results showed that lesions of cholinergic neurons in the HDB produced depressive-like and anxiety-like behaviors, such as anhedonia, increased immobility time in the FST, decreased locomotor activity, memory deficit in the contextual fear conditioning, and reduced time in open arms in the EPM. These depressive-like behaviors but not anxiety-like behavior could be reversed by repeated treatment with FLX or with an acute dose of ketamine. These results clearly demonstrate that cholinergic neurons in the HDB play an important role in the production of depressive-like behaviors.

Sucrose preference is typically reduced following chronic stress exposure. Such a reduction in sucrose preference is described as anhedonia (Willner et al., 1992). The FST is often used as a method of measuring the efficacy of antidepressants in rodents. Immobility is presumed to reflect a state of behavioral despair and depression (Slattery and Cryan, 2012). There are several stress factors such as single housing after surgery, a battery of behaviors tested, should be considered in the experiments. It has been demonstrated that isolated rats exhibit locomotor hyperactivity in a novel environment and increased anxiety in several paradigms (Weiss et al., 2004), on the other hand, social isolation did not affect open-field activity, but increased depressive-like behavior (Brenes et al., 2008). Here, the depressive-like behaviors tested by using SPT and FST and anxiety-like behavior by EMP produced after lesion of HDB cholinergic neurons. Conversely, locomotion activity was decreased after lesion of HDB neurons, which is differentiate from hyperactivity effects of the isolation stress. In fact, there is emerging evidence that increased acetylcholine levels in hippocampus reduce anxiety (Degroot and Treit, 2002). Additionally, three independent tests of SPT across the experiments were used on 15, 17, and 25 days, the deteriorated effects of sucrose preference were not observed after long-term housing singly. Thus, the present results indicated that stress response might partly contribute to depressive-like and anxiety-like behaviors, but it is not the key factor in the present study.

The hippocampus in mammals receives massive input from cholinergic neurons in the basal forebrain. Our specific lesion of cholinergic neurons in the HDB also caused neuronal loss in the entire hippocampus, which is consistent with previous reports (Mesulam et al., 1983; Rye et al., 1984; Salehi et al., 2006). Recent evidence has been shown that cholinergic degeneration in the medial septal area induced by intracerebroventricular administration of 192 IgG-saporin results in an increase in the number of microglial cells and neuron degeneration in the dorsal hippocampus (Dobryakova et al., 2019). Now, we used both non-trans-synaptic rabies viruses and fluorogold retrograde tracers to observe the projection of HDB to hippocampus. The results showed the direction projection from HDB to hippocampus CA1 (Shown in the Supplementary Material). Moreover, the context dependent freezing time in the fear conditioning was decreased after introducing a lesion of the HDB in the present study. Contextual fear conditioning is a hippocampus-dependent learning task, and the dentate gyrus (DG) and CA1 of rats are responding to association learning during the fear conditioning (Gilmartin and McEchron, 2005; Maren, 2008). The deficit of contextual memory might be correlated with neuron loss in DG and CA1 within hippocampus after injection of 192 IgG-saporin into HDB, it would be necessary to measure relationship between a neuron loss marker such as apoptosis in the hippocampus and memory or other behaviors. Thus, the results suggested that selectively loss of cholinergic neurons in HDB and pyramidal neurons within hippocampus not only produces the deficit of cognitive functions, but also results in depressive-liked and anxiety-like behaviors.

Neuron atrophy caused by chronic stress exposure in the rodent hippocampus and prefrontal cortex results in depressive-like behavior (McEwen, 1999; Vyas et al., 2002; Banasr et al., 2007). In the present study, cell loss was observed in the hippocampus. One potential mechanism underlying pathogenesis of depression induced by lesion of HDB and hippocampus may be accounted for impaired cholinergic input to the hippocampus. Both pharmacological and molecular genetic decreases in hippocampal AChE activity increase depression-like behaviors that is sensitive to FLX treatment (Mineur et al., 2013). Second, the selective loss of cholinergic cells is accompanied with microglial proliferation and activation of astrocytes in hippocampus (Rossner et al., 1995; Dobryakova et al., 2019), and activated microglia and astrocytes and elevated levels of proinflammatory cytokines may be involved in the pathogenesis of depression following a lesion of HDB (Singhal and Baune, 2017).

In the present study, we used two typical antidepressants such as FLX or ketamine to test the reversal effects on the depressive-like behaviors induced by lesion of HDB. Daily administration of FLX at dose of 10 mg/kg could significantly increase the sucrose preference for 3 or 11 days, and increase immobility in rats forced to swim for 9 days. The results are consistent with the previous studies that repeated administrations of FLX improve certain aspects of depression-like behavior and hippocampal functions (David et al., 2009; Siopi et al., 2016). It has been shown that 21-day treatment with fluoxetine at 1 mg/kg reduce the immobility in the FST (Contreras et al., 2001; Rodriguez-Landa et al., 2018). The behaviors such as sucrose preference and immobility in FST improved more quickly may account for using a relatively high dose of FLX in the present experiment. Acute treatment with ketamine could improve the sucrose preference on 1 or 3 day and reverse the immobility time in the FST paradigm on 9 day in the HDB lesioned rats. The antidepressant effect is identical to previous reports that ketamine can take fast-acting antidepressant and last near 1 week (Autry et al., 2011). Chronic FLX treatment activate adult neurogenesis in the dentate gyrus of the hippocampus, known to counteract depression (Micheli et al., 2018). Lower expression AMPAR, impaired mTOR complex signaling pathway, and lower level of brain-derived neurotrophic factor (BDNF) is linked to neuronal atrophy and depression (Corriger and Pickering, 2019). Ketamine has a rapid antidepressant effect in animal models with increases of AMPAR activity, levels of phosphorylated mTOR, and expression of BDNF (Ignacio et al., 2016). Thus, we concluded that the depressive-like behavior induced by lesion of cholinergic neurons in HDB could be reversed by FLX or ketamine administration.

However, repeated administrations of FLX or acute treatment with ketamine failed to inhibit the anxiety-like behavior but increased the locomotion activity in the HDB lesioned rats. A number of studies have been shown that chronic administration with FLX also inhibits the anxiety-like behavior after chronic social isolation (Todorovic and Filipovic, 2017), whereas acute treatment of FLX induces the anxiogenic profile and chronic FLX treatment has limited anxiolytic effects (Silva and Brandao, 2000). Meanwhile, a proof-of-concept trial provides initial evidence that ketamine may be effective in reducing anxiety (Taylor et al., 2018). One explanation for these conflicts lies in the fact that depression or anxiety underlying neurobiological substrates is different after lesion of cholinergic neurons in HDB. The discrepancy between locomotion activity and anxiolytic-like effects of fluoxetine or ketamine in this model deserves further study. There are also several limitations and caveats in the present study that should be pointed out. First, we failed to quantitate the lesion of hippocampus neurons and neuron loss markers and to analyze the possible damage of other area in the brain after injection of 192 IgG-saporin into HDB. Second, the sex of the animal used had not been considered. Many studies of depression have shown the sex differences in animal (Sanchez et al., 2018) or depression patients (Rubinow and Schmidt, 2019), now the generalizability of these results to females is not obvious. Finally, here we did not observe the morphological change of hippocampus and neurogenesis in hippocampus after chronic treatment with FLX, whether its antidepressant interventions and improved behavioral recovery after HDB lesion correlates with amelioration of hippocampal pathology is warranted to further study.

In conclusion, FLX and ketamine reversed depression-like states as expected, however, anxiety-like states were not restored. Thus, the behaviors induced by introducing a lesion of the cholinergic neurons in the HDB are consistent with the behavioral expression of depression in humans and can be quantified. Moreover, both FLX and ketamine are effective in the treatment of MDD (Basso et al., 2020; Mayberg et al., 2000). Our results are the first to demonstrate that a lesion of the cholinergic neurons in the HDB in the basal forebrain produces depressive-like behaviors, which suggests that the cholinergic neurons that project from the HDB to the hippocampus may play an important role in the production of depression.

## Data Availability Statement

The raw data supporting the conclusions of this article will be made available by the authors, without undue reservation, to any qualified researcher.

## Ethics Statement

The animal study was reviewed and approved by the Ningbo University Ethics Committee of Animals.

## Author Contributions

LC, YK, and HM performed the research, analysis of the data, and writing of the manuscript. LG, YZ, and HZ performed the research. HL and FZ were responsible for guarantee of the animal and experimental conditions. WZ was responsible for the study design and revising the manuscript. All authors contributed to the article and approved the submitted version.

## Conflict of Interest

The authors declare that the research was conducted in the absence of any commercial or financial relationships that could be construed as a potential conflict of interest.
