# Supplementary material for: Fluoxetine and Ketamine Reverse the Depressive but Not Anxiety Behavior Induced by Lesion of Cholinergic Neurons in the Horizontal Limb of the Diagonal Band of Broca in Male Rat
Source: Front Behav Neurosci. 2021 Feb 18;15:602708. doi: 10.3389/fnbeh.2021.602708 (PMC7930217; doi:10.3389/fnbeh.2021.602708)
Supplement: Supplementary file 1 [file Table_1.DOCX]

**Projection of horizontal limb of the diagonal band of Broca to hippocampus CA1 projections by both Retrograde tracers**

**Experiment 1: Projection of HDB to hippocampus CA1 projections by RV-dG-GFP**


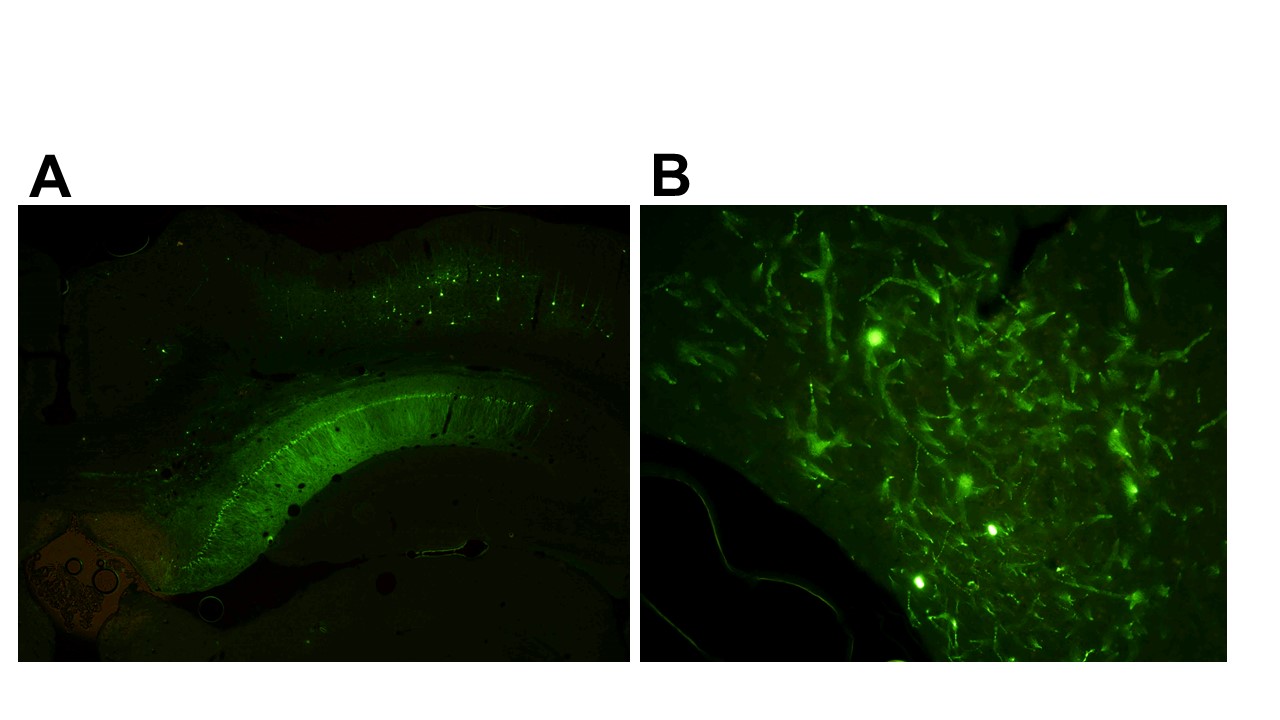
These rats were anesthetized with sodium pentobarbital (50 mg/kg, i.p.) and positioned into a stereotaxic apparatus (Stoelting 51950, USA). Non-trans-synaptic rabies viruses (RV-dG-GFP, 1 μl per side, 3.8×10^8^ V.G./mL, Brain VTA Inc., Wuhan, China) were used to trace horizontal limb of the diagonal band of Broca (HDB)-hippocampus CA1 projections. The viruses were injected into the CA1 (AP: 3.8 mm; ML: 2.0 mm; DV: 3.5 mm) by using glass micropipettes which driven by a syringe pump at a speed of 0.1 μL min^−1^. The animals were individual housed in an isolated room for 9 d. The animals were then anesthetized and perfused with saline followed by 4% PFA. Brains were taken from the animals, and placed in 4% PFA overnight, and immersed in 30% sucrose in PBS. The brains were sectioned on a Cryostat Microtome (Leica CM1850, Germany) in the coronal plane at a thickness of 30 μm. Viruses expression was observed by fluorescent microscopy (Olympus BX 51, Japan).

**Fig. S1. Observation of projection from HDB to hippocampus by RV-dG-GFP tracer.** Infusion of RV-dG-GFP into the hippocampus. (A) Location of injection in the hippocampus. (B) The positive expression of virus GFP in the soma and axon in the HDB.

**Experiment 2: Projection of HDB to hippocampus CA1 projections by** **retrograde** **fluorogold tracer**

These rats were anesthetized with sodium pentobarbital (50 mg/kg, i.p.) and positioned into a stereotaxic apparatus (Stoelting 51950, USA). Retrograde fluorogold tracer (FG, a retrograde tracer, 4%, 0.4 μl per side) was used to trace HDB-hippocampus CA1 projections. The FG was injected into the CA1 (AP: 3.8 mm; ML: ±2.0 mm; DV: 3.5 mm) by using glass micropipettes which driven by a syringe pump at a speed of 0.1 μL min^−1^. The animals were individual housed in an isolated room for 3 d. The animals were then anesthetized, and perfused with saline followed by 4% PFA. Brains were taken from the animals, and placed in 4% PFA overnight, and immersed in 30% sucrose in PBS. The brains were sectioned on a Cryostat Microtome (Leica CM1850, Germany) in the coronal plane at a thickness of 30 μm. FG expression was observed by fluorescent microscopy (Olympus BX 51, Japan).

**
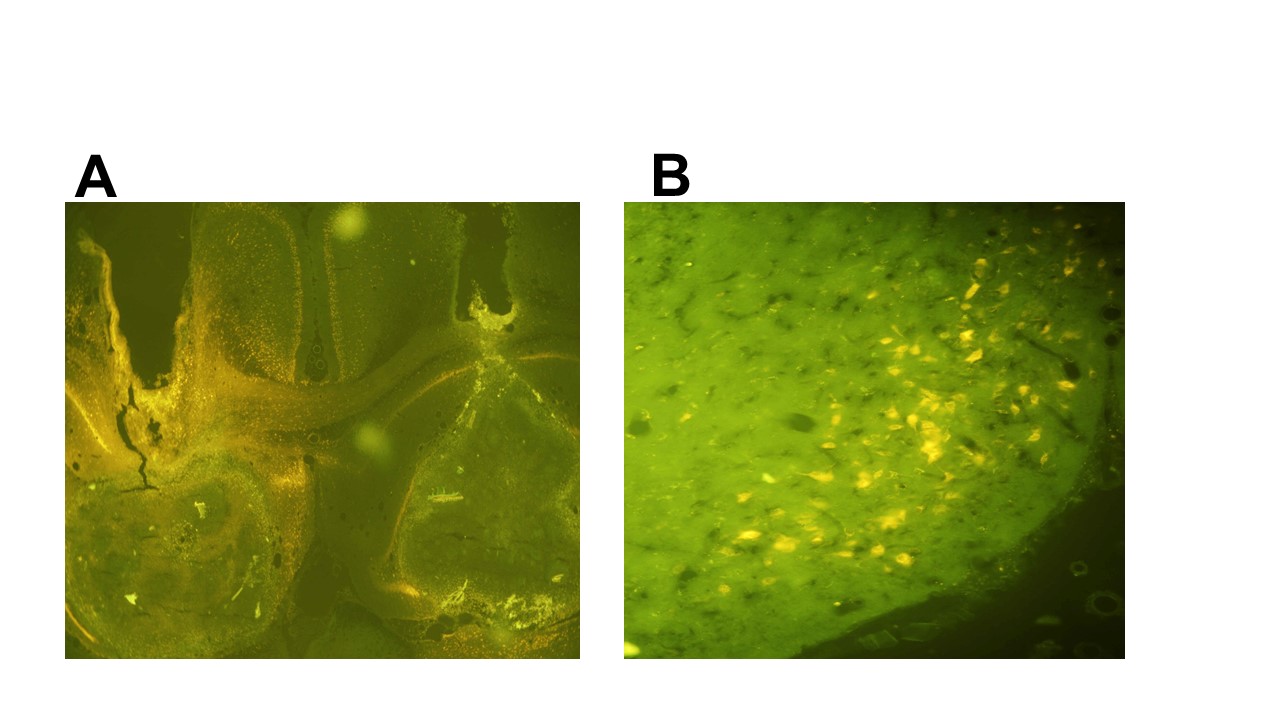
**

**Fig. S2. Observation of projection from HDB to hippocampus by retrograde fluorogold tracer.** Infusion of retrograde fluorogold tracer into the hippocampus. (A) Location of injection in the hippocampus. (B) The positive expression of retrograde fluorogold in the neurons in the HDB.
